# Creation of Resveratrol-Enriched Rice for the Treatment of Metabolic Syndrome and Related Diseases

**DOI:** 10.1371/journal.pone.0057930

**Published:** 2013-03-04

**Authors:** So-Hyeon Baek, Woon-Chul Shin, Hak-Seung Ryu, Dae-Woo Lee, Eunjung Moon, Chun-Sun Seo, Eunson Hwang, Hyun-Seo Lee, Mi-Hyun Ahn, Youngju Jeon, Hyeon-Jung Kang, Sang-Won Lee, Sun Yeou Kim, Roshan D’Souza, Hyeon-Jin Kim, Seong-Tshool Hong, Jong-Seong Jeon

**Affiliations:** 1 National Institute of Crop Science, Rural Development Administration, Iksan, Chonbuk, Korea; 2 Graduate School of Biotechnology, Kyung Hee University, Yongin, Gyeonggi, Korea; 3 Crop Biotech Institute, Kyung Hee University, Yongin, Gyeonggi, Korea; 4 Graduate School of East-West Medical Science, Kyung Hee University, Yongin, Gyeonggi, Korea; 5 Laboratory of Genetics and Department of Microbiology, Chonbuk National University Medical School, Jeonju, Chonbuk, Korea; 6 Department of Plant Molecular Systems Biotechnology, Kyung Hee University, Yongin, Gyeonggi, Korea; 7 College of pharmacy, Gachon University, Incheon, Korea; 8 BDRD Research Institute, JINIS Biopharmaceuticals Inc., Wanju, Chonbuk, Korea; TGen, United States of America

## Abstract

Resveratrol has been clinically shown to possess a number of human health benefits. As a result, many attempts have been made to engineer resveratrol production in major cereal grains but have been largely unsuccessful. In this study, we report the creation of a transgenic rice plant that accumulates 1.9 µg resveratrol/g in its grain, surpassing the previously reported anti-metabolic syndrome activity of resveratrol through a synergistic interaction between the transgenic resveratrol and the endogenous properties of the rice. Consumption of our transgenic resveratrol-enriched rice significantly improved all aspects of metabolic syndrome and related diseases in animals fed a high-fat diet. Compared with the control animals, the resveratrol-enriched rice reduced body weight, blood glucose, triglycerides, total cholesterol, and LDL-cholesterol by 24.7%, 22%, 37.4%, 27%, and 59.6%, respectively. The resveratrol-enriched rice from our study may thus provide a safe and convenient means of preventing metabolic syndrome and related diseases without major lifestyle changes or the need for daily medications. These results also suggest that future transgenic plants could be improved if the synergistic interactions of the transgene with endogenous traits of the plant are considered in the experimental design.

## Introduction

Resveratrol (3,5,4′-trihydroxy-trans-stilbene) is a non-flavonoid polyphenol-type stilbene compound found in several fruits and vegetables. Although resveratrol has various beneficial health effects, its effect on metabolic syndrome is the best characterized [1]. Since this finding, a major research objective has been to create transgenic plants that accumulate resveratrol. The transfer of stilbene synthase (*STS*) genes has been previously accomplished in a number of plants [2]. Of these transgenic plant studies, however, reasonable levels of resveratrol production were only observed in a few cases, including transgenic tobacco [3], [4], tomato [5], and lettuce [6]. Notably, resveratrol production has not been successfully achieved in human-edible agronomically significant crops such as cereal grains. A grain crop plant with proven activity against metabolic syndrome is therefore an ideal target for resveratrol production, considering that metabolic syndrome and related diseases could be controlled by dietary intake.

The *Oryza sativa* japonica variety Dongjin (Dongjin rice), developed by the Rural Development Administration of Korea, yields a grain that is rich in fiber and in polyphenols that confer low levels of anti-metabolic syndrome activity [7]. It is thus reasonable to assume that a transgenic Dongjin rice strain that expresses resveratrol may prevent and treat metabolic syndrome and related diseases through a synergistic effect of its innate and transgenic properties. To test this hypothesis, we generated transgenic resveratrol-enriched rice and assessed its efficacy in controlling metabolic syndrome and related diseases in a mouse model.

## Results and Discussion

### Production of Transgenic Rice

We cloned the resveratrol biosynthesis gene, stilbene synthase (*STS*), from the peanut *Arachis hypogaea* variety Palkwang, a well-known plant species that contains high quantities of resveratrol [8]. Sequence analysis of the cloned cDNA, designated *AhSTS1* (GenBank accession no. DQ124938), showed a high similarity to previously identified *STS*s (Figure S1). In the peanut, *STS* appeared to be highly expressed in the early and middle stages of the developing pods after flowering but not in the leaves (Figure S2). To determine whether *AhSTS1* encodes a functional STS enzyme, we cloned the *4-coumaroyl-CoA ligase (4CL)* gene from *Arabidopsis thaliana* (*At4CL2*). The product of this gene converts *p*-coumaric acid into *coumaroyl* **-** *CoA by* coupling it with a coenzyme. We reasoned that the coexpression of *AhSTS1* and *At4CL2* might lead to resveratrol production using *p*-coumaric acid and malonyl-CoA [9], [10]. *AhSTS1* and *At4CL2* were cotransformed into *E. coli*, and the production of the recombinant AhSTS1 and At4CL2 proteins was confirmed using western blot analysis with anti-His and anti-MBP antibodies, respectively (Figure S3). GC-MS analysis of the culture grown in medium supplemented with *p*-coumaric acid demonstrated that one fraction eluted by HPLC was identical to the resveratrol standard (Figure S4). This finding establishes AhSTS1 as an active STS enzyme. In contrast, cells transformed with control vectors did not produce resveratrol.

Several transgenic cereal plants have been produced with the aim of accumulating an adequate quantity of resveratrol in the edible portion of cereal crops [11], [12]. However, these transgenic cereal plants failed to accumulate resveratrol in the grain, likely because of unfavorable chimeric constructs or because the foreign gene was inserted into a chromosomal locus that was unfavorable for expression. In this study, we constructed a chimeric fusion between the maize *Ubiquitin1* (*Ubi1)* promoter, which produces high levels of activity in monocots [13] and *AhSTS1* to express *AhSTS1* in rice. Then, we conducted phenotypic expression analysis at each step before proceeding to the next step to confirm the proper expression of the transgene during the creation of our transgenic rice. We transferred the chimeric construct into embryonic calli induced from the mature embryo of Dongjin rice using the *Agrobacterium*-mediated transformation method to generate transgenic calli. Somatic embryos formed from the transgenic calli were germinated on N6 medium containing phosphinothricin (PPT) to regenerate small plantlets. HPLC analysis of the metabolite profiles of the T_1_ seeds produced by 398 plants showed that 129 T_1_ lines of Dongjin rice produced over 0.1 µg resveratrol per gram of seed.

**Figure 1:**
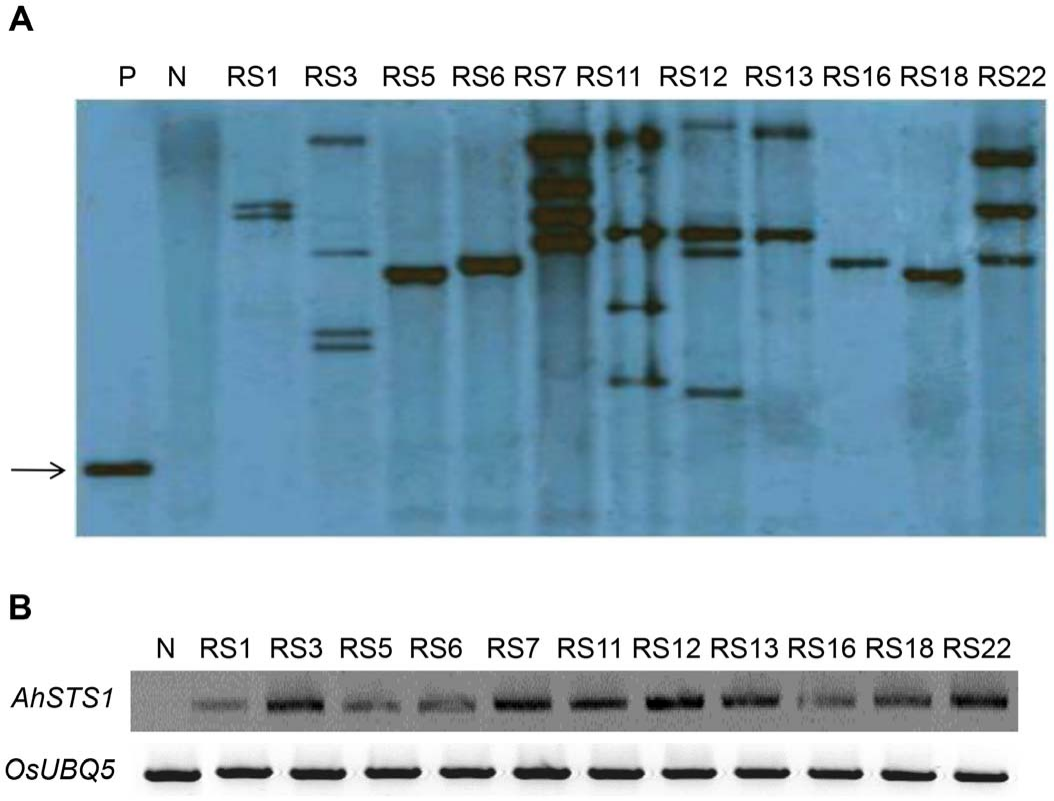
Molecular characterization of transgenic rice lines expressing *AhSTS1*. (A) Southern blot analysis. Genomic DNA in lanes P and RS1 to RS22 were digested with *Bam*HI (specific to the T-DNA region). The arrow indicates the fragment (1.2 kb) hybridized with the *AhSTS1* cDNA probe. P, pSB2220 vector; N, non-transgenic wild-type Dongjin; lanes RS1 - RS22, representative transgenic Dongjin lines out of 129 T_1_ samples. (B) RT-PCR analysis. Total RNA from leaf samples of the same lines as in (A) was analyzed. *OsUBQ5* was included as a PCR control.

We planted all of the 129 T_1_ seeds in a paddy field to enable a thorough analysis of agricultural traits. Southern blot analysis demonstrated that these transgenic rice lines carried one to four copies of the transgene (Figure 1A), and RT-PCR analysis indicated that all of the transgenic lines exhibited high levels of *AhSTS1* expression (Figure 1B). In the rice paddy field, many of the transgenic plants displayed partial sterility at the flowering stage. However, several lines were completely fertile. A similar infertility phenotype was observed in tobacco expressing high levels of an *STS* gene [4], suggesting that transgenic overexpression of *STS* affects the fertility of plants.

### Resveratrol Analysis of Transgenic Rice

**Figure 2:**
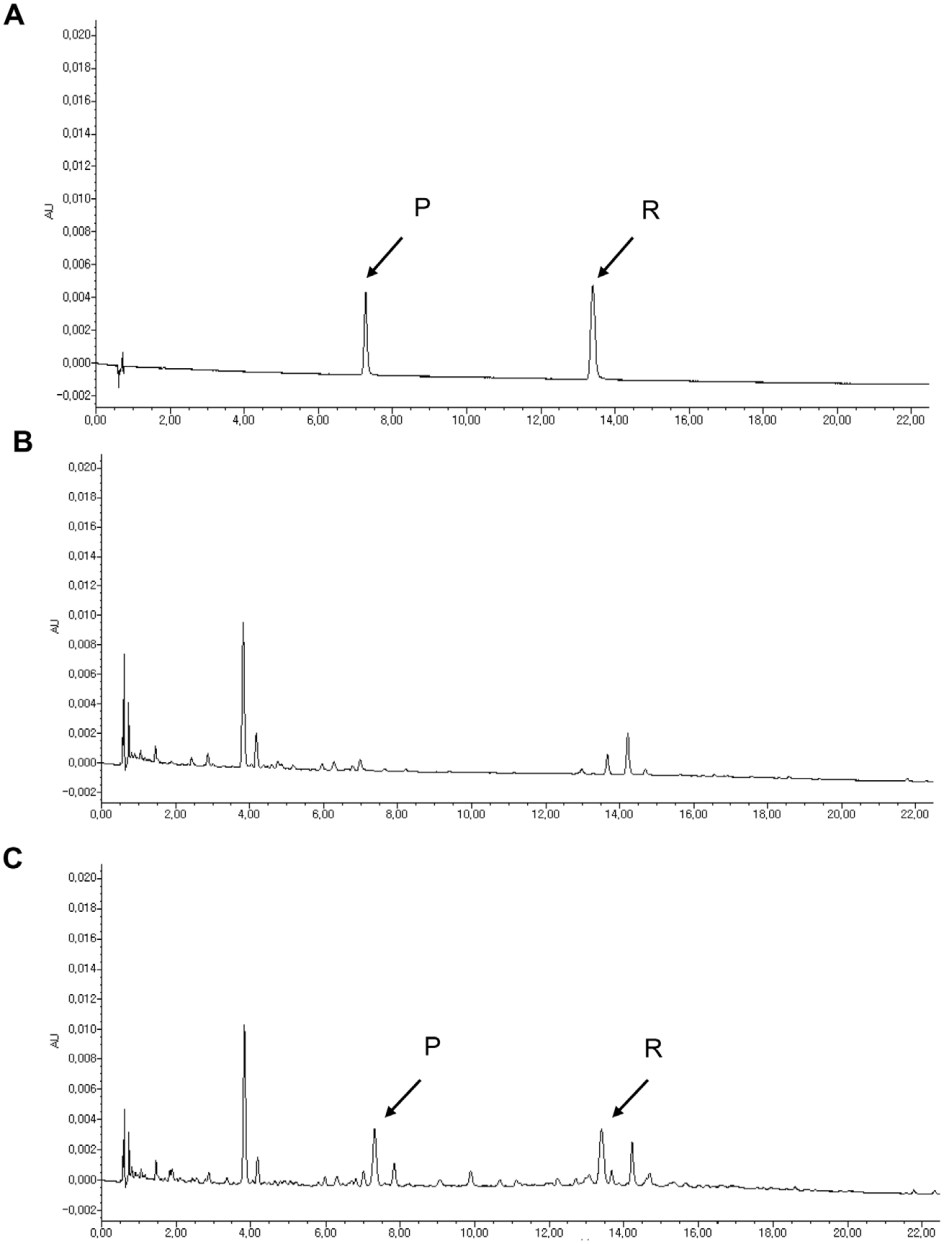
The identification of resveratrol and piceid in the grains of wild-type Dongjin and transgenic rice using HPLC. (A) A standard mixture of piceid (P) and resveratrol (R). (B) Wild-type Dongjin rice. (C) Transgenic Dongjin rice RS18. The arrows indicate the positions of piceid (P) and resveratrol (R).

**Figure 3:**
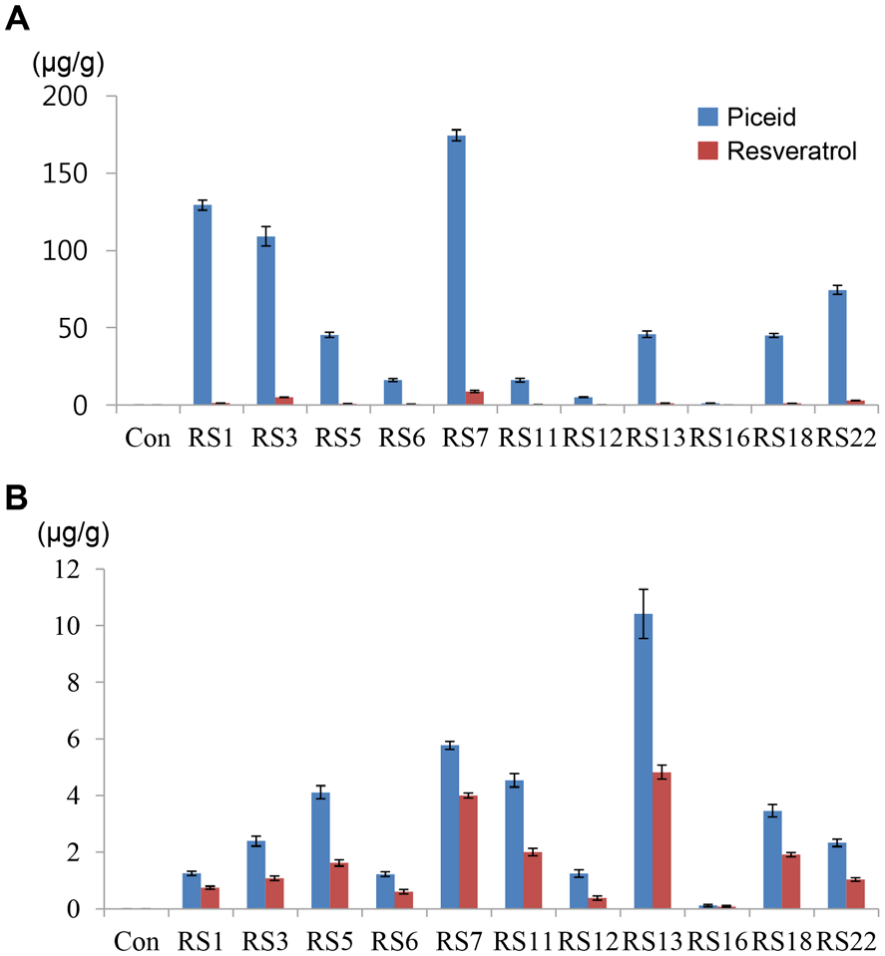
The quantification of the piceid and resveratrol levels in the leaves and grains of wild-type Dongjin rice and representative transgenic Dongjin rice lines out of 398 T_1_ samples. (A) Leaves. (B) Grains.

To assess the biosynthetic profile of the transgene in Dongjin rice, we analyzed resveratrol and the related resveratrol glucoside piceid from all tissues of the transgenic rice plants using HPLC. The health benefits of piceid are less than resveratrol [14], [15]. In the wild-type Dongjin rice, HPLC analysis failed to detect resveratrol or piceid (Figure 2B). In the leaves of the transgenic rice plant, however, we detected high levels of piceid ranging from 1.2–174.4 µg/g and low levels of resveratrol ranging from 0–8.9 µg/g (Figure 3A). On the other hand, the grains of the transgenic rice contained comparable levels of resveratrol (0.1–4.8 µg/g) but a relatively low quantity of piceid (0.1–10.4 µg/g) compared with the corresponding levels in the leaves (Figure 2C and 3B). These quantities in the grain of the transgenic rice are similar to the levels of resveratrol (0.8–5.8 µg/mL) reported in high-quality red wine [16]. Based on agricultural, biochemical, and genetic traits, we chose the homozygous transgenic line RS18 as a candidate strain for further experiments. The RS18 line contained a single transgene copy and exhibited a relatively high expression level of the transgene among all of the transgenic lines produced; its performance with respect to agronomic traits was similar to that of the parental Dongjin rice (Table S1).

**Figure 4:**
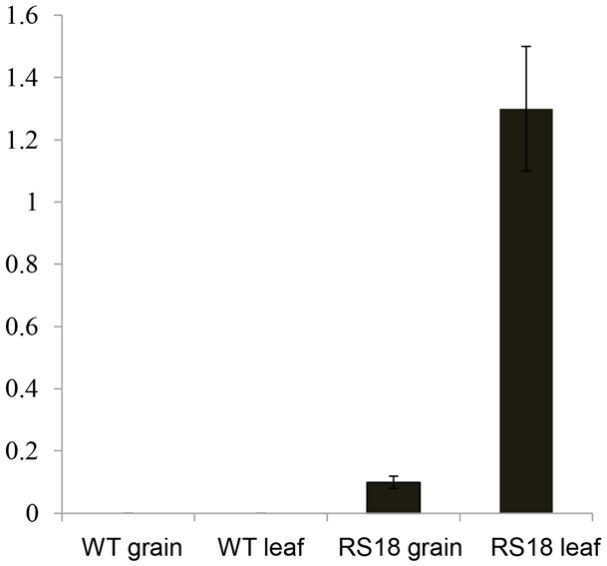
Measurement of resveratrol-specific glucosyltransferase activity in rice leaves and grains. WT, wild-type Dongjin rice; RS18, *AhSTS1* transgenic Dongjin rice line. Remarkably high resveratrol-specific glucosyltransferase activity was found only in the leaves of the transgenic rice line RS18.

As mentioned above, the grains of the transgenic plants, including RS18, contained a relatively high quantity of resveratrol compared with piceid, whereas the reverse was observed in the leaves. This unequal distribution of the two metabolites could be due to glucosyltransferase activity. Glucosyltransferase activity is known to be involved in the formation of piceid from resveratrol [17], [18]. The relatively high level of piceid in the leaves suggested that the leaves might have higher glucosyltransferase activity than the grains. Quantification of glucosyltransferase activity in wild type and RS18 rice showed that the RS18 leaves exhibited much higher resveratrol glucosyltransferase activity than the grains. The wild-type leaves and grains did not show any resveratrol glucosyltransferase activity (Figure 4). This observation suggests that resveratrol-specific glucosyltransferase activity is induced in response to resveratrol production in the transgenic plant. Further study is necessary to identify the gene(s) responsible for this resveratrol glucosyltransferase activity.

Rice seeds are milled to create polished white rice, which is then consumed. We compared the resveratrol levels of unpolished brown grains and polished white grains of the RS18 strain. The results indicated that the unpolished and polished grains contained similar levels of resveratrol, 1.9 and 1.7 µg/g, respectively (Table S2). This finding suggests that most of the resveratrol accumulates in the endosperm rather than in the other tissues, such as the aleurone layer and the embryo.

### Assessment of Anti-metabolic Syndrome Activity of Transgenic Resveratrol-enriched Rice

**Figure 5:**
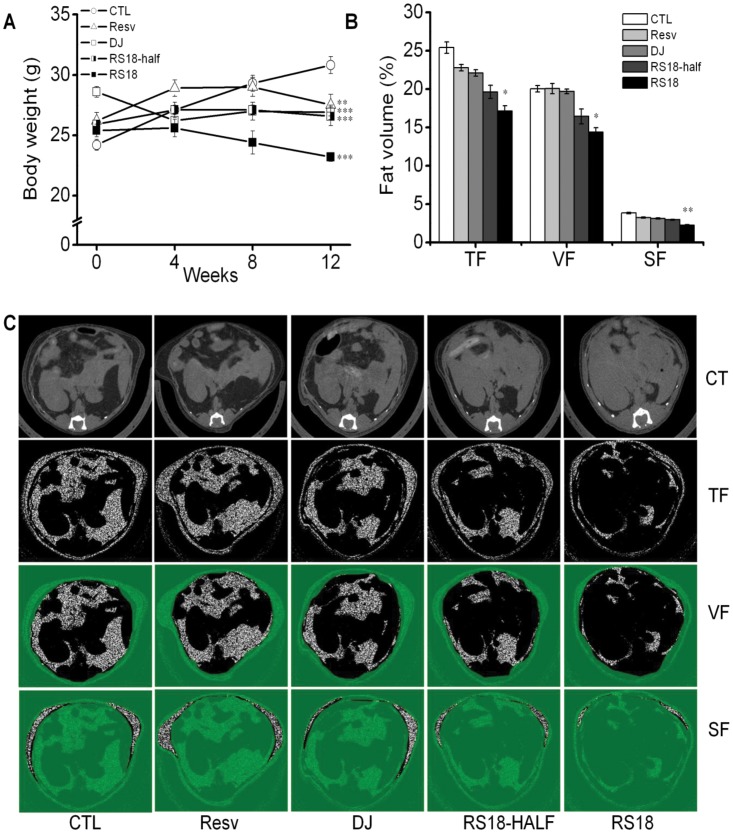
The effects of the resveratrol-enriched rice on body weight and body fat volume. (A) The body weight of mice during a 12-week period. The values represent the means ± SEM (n = 16). An unpaired Student’s t-test was used for the statistical analysis; *p<0.05, **p<0.01, ***p<0.001 compared with CTL. (B) The fat volume of mice using *in vivo* micro-CT image analysis. (c) Representative images of micro-CT and fat area. The values represent the means ± SEM (n = 5). TF, total fat; VF, visceral fat; SF, subcutaneous fat (SF).

We examined the efficacy of the transgenic resveratrol-enriched rice on metabolic syndrome and on related diseases associated with the levels of blood glucose, triacylglycerol, total cholesterol, LDL cholesterol and HDL cholesterol using an *in vivo* mouse model. We assessed in the mice whether the innate characteristics of the rice and the transgenic resveratrol had a synergistic effect that boosts anti-metabolic syndrome activity. C57BL inbred mice were fed a high-fat diet (HFD) for 12 weeks to induce metabolic syndrome and related diseases. The food consumption rate was the same among different mouse groups (each individual mouse consumed 4 g of food per day). The diet-induced obesity mice were fed the HFD in the control group or a modified HFD in the experimental group, in which the carbohydrate source was replaced with the resveratrol-enriched rice (Table S3). We periodically monitored the changes in the blood profiles and body weight in each mouse group under continuous HFD conditions (Table 1 and Figure 5). Compared with the HFD control, supplementation with resveratrol at the same level as that produced by RS18 resulted in modest improvement, consistent with previous reports on the effects of resveratrol on metabolic syndrome and related diseases [1], [19], [20]. The consumption of Dongjin rice also resulted in a similar improvement in lipid profile and blood glucose levels, as expected due to its endogenic nature. Notably, the consumption of the resveratrol-enriched Dongjin rice significantly improved all aspects of metabolic syndrome and related diseases, lowering the blood glucose by 22.0%, triacylglycerol by 37.4%, total cholesterol by 27.0%, and LDL cholesterol by 59.6%, whilst increasing the HDL cholesterol by 14.8% (RS18 compared with the HFD control). An RS18-half group with a modified HFD, in which only half the amount of corn starch was replaced by RS18 rice, failed to have an effect similar to that observed in the RS18 group, indicating a dose-dependent effect of the resveratrol-enriched rice. As expected from the blood profiles, body weights were greatly reduced in mice fed the resveratrol-enriched rice (RS18 group; 24.7% compared with the control) and was different from the other treatments (the resveratrol supplementation group, Dongjin rice group, and RS18-half group) (Figure 5A). Micro-CT image analysis of abdominal fat deposition showed that the total, visceral, and subcutaneous fat volumes in the resveratrol-enriched rice group (RS18) were 21.55%, 16.33%, and 3.10%, respectively, which were significantly lower than the fat volumes from the HFD control (25.43%, 20.02%, and 3.83%, respectively) (Figure 5B). Representative images clearly indicated that the total, visceral and subcutaneous fat accumulation volumes were lowest in the RS18 group compared with the other treatments (Figure 5C).

**Table 1:** The effects of the resveratrol-enriched rice on blood lipid and glucose levels.

	Week 0	Week 4	Week 8	Week 12
**TG (mg/dL)**				
**CTL**	84.5±17.6	86.4±19.7	81.8±6.2	81.5±22.7
**Resv**	84.3±20.7	78.9±28.9	64.4±13.9^b^	60.2±11.9^b^
**DJ**	85.5±19.3	70.2±25.7^a^	65.5±20.9^b^	64.7±19.3^b^
**RS18-half**	83.1±18.9	75.4±19.1	60.5±6.6^b^	64.3±14.7^b^
**RS18**	82.2±15.6	60.6±10.9^b^	60.5±14.6^b^	51.0±11.3b,c
**TC (mg/dL)**				
**CTL**	178.5±41.8	177.3±29.7	196.0±15.3	198.3±30.7
**Resv**	180.9±37.8	170.3±26.6	172.7±11.2^b^	157.3±4.2^b^
**DJ**	178.4±27.8	171.5±39.2	170.1±23.5^b^	167.2±21.3^b^
**RS18-half**	177.4±32.4	160.0±26.9	163.4±9.7^b^	159.7±5.9^b^
**RS18**	181.8±24.4	157.1±20.0^a^	159.4±14.4^b^	144.7±12.2b,d
**HDL-C (mg/dL)**				
**CTL**	82.7±5.6	85.9±19.6	85.0±8.9	83.4±6.9
**Resv**	84.0±4.1	89.4±24.1	99.5±3.4^b^	94.4±12.5^b^
**DJ**	82.3±8.8	86.5±17.9	86.7±8.7	89.2±12.1
**RS18-half**	84.5±8.0	78.8±14.3	84.7±7.0	89.3±11.3
**RS18**	78.4±7.2	85.4±5.2	95.3±6.6b,c	97.9±8.6 b,c
**LDL-C (mg/dL)**				
**CTL**	78.4±43.1	74.2±31.6	93.8±11.2	90.8±15.1
**Resv**	85.9±36.1	65.1±35.8	60.3±15.1^b^	50.8±6.8^b^
**DJ**	79.1±29.4	70.9±20.1	70.3±18.9^b^	65.1±12.3^b^
**RS18-half**	76.3±30.2	66.1±29.9	66.6±10.1^b^	57.5±6.7b,c
**RS18**	87.0±23.7	59.6±19.9	51.9±20.1b,c	36.6±10.6b,d
**Glucose (mg/dL)**				
**CTL**	193.1±22.7	201.7±60.4	233.7±25.0	236.3±23.2
**Resv**	195.2±18.8	201.4±20.9	205.1±24.4^b^	203.3±18.5^b^
**DJ**	194.5±23.2	203.3±27.8	208.1±24.1^b^	207.4±19.1^b^
**RS18-half**	194.4±24.7	198.1±23.8	202.4±23.3^b^	200.4±19.4^b^
**RS18**	192.1±28.2	194.0±21.0	187.3±24.0b,c	184.3±18.3b,d

The values represent the means ± SEM (n = 16). CTL, mice fed a HFD (D12451); Resv, mice fed a HFD supplemented with resveratrol; DJ, mice fed a HFD in which the corn starch and sucrose were replaced with Dongjin rice; RS18-half, mice fed a HFD in which half of the corn starch and sucrose were replaced with the resveratrol-enriched rice; RS18, mice fed a HFD in which the corn starch and sucrose were replaced with the resveratrol-enriched rice. Values in a column with a superscripted letter indicate statistical significance as analyzed by an unpaired Student’s t-test;

a p<0.05 compared with CTL;

b p<0.01 compared with CTL;

c p<0.05 compared with DJ;

d p<0.01 compared with DJ.

The most important finding from this experiment was the synergistic effect of Dongjin rice and transgenic resveratrol in the RS18 group compared with treatment by resveratrol supplementation or Dongjin rice alone. The resveratrol-enriched Dongjin rice, RS18, was thus found to be as effective at treating metabolic syndrome and related diseases as typical pharmaceutical drugs for these disorders in reducing the blood glucose, LDL/total cholesterol, or body weight. Hence, resveratrol-enriched rice is a potentially feasible and viable choice to treat most, if not all, aspects of metabolic syndrome and related diseases.

**Figure 6:**
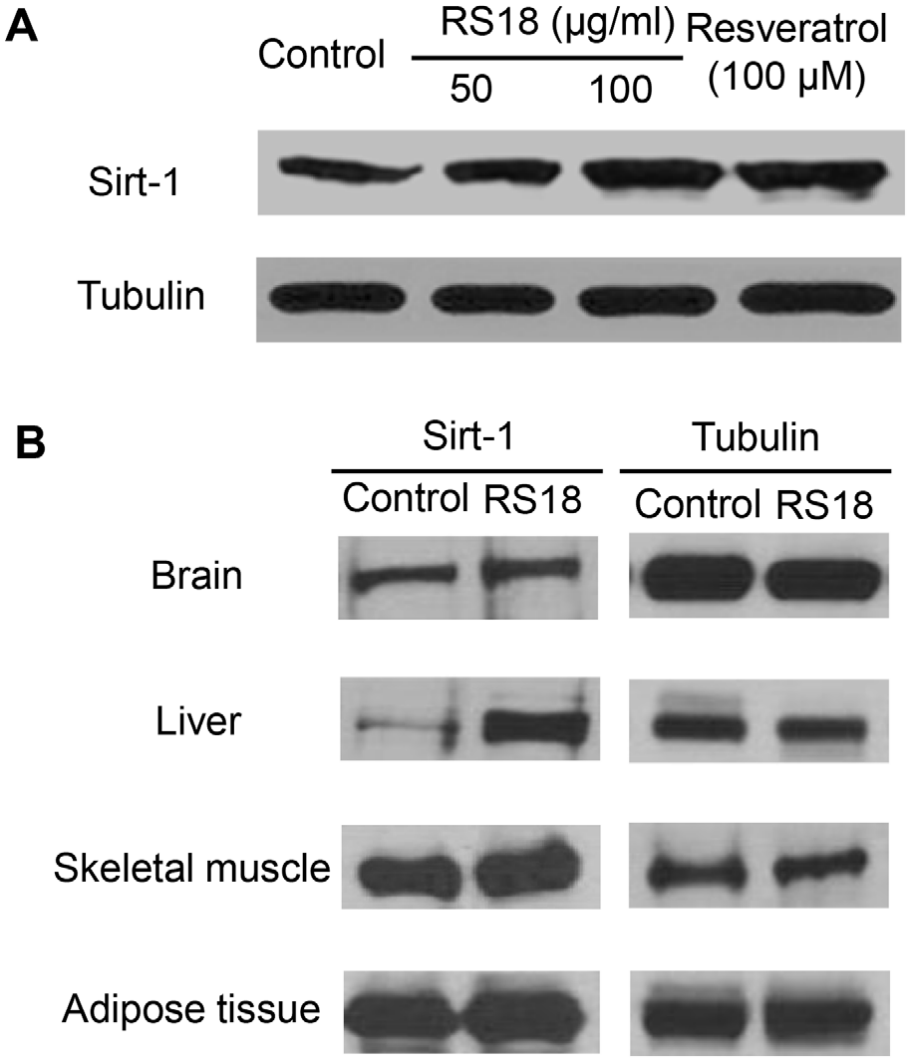
The effects of the resveratrol-enriched rice on Sirt1 protein levels in cells and in mice. (A) The level of Sirt1 protein in SH-SY5Y cells. The SH-SY5Y cells were treated with 70% ethanol extracts of RS18 transgenic grain (50 and 100 µg/mL) or resveratrol (100 µM) for 24 h. (B) Mice treated with RS18 transgenic grain. The organs, including the brain, liver, skeletal muscle and adipose tissues, were harvested from mice that had been fed RS18 transgenic grain. Subsequently, 30 µg of protein from each lysate was used for western blot analysis. Equal protein loading was confirmed using an anti-tubulin antibody.

The central nervous system controls nutrient levels in an effort to maintain metabolic homeostasis through the feedback and crosstalk of many organs [21]. In the brain, Sirt1, a nicotinamide adenine dinucleotide (NAD^+^)-dependent deacetylase, is a key regulator of the energy homeostasis involved in glucose and lipid metabolism [22]–[24]. To examine the effect of transgenic rice grains on the level of Sirt1 protein, we treated human neuroblastoma SH-SY5Y cells with ethanol extracts from the grains of RS18 (50 and 100 µg/mL). Western blot analysis indicated that the levels of Sirt1 protein were higher in the treated cells than in untreated cells. Similar increases in Sirt1 protein were observed in cells treated with 100 µM resveratrol (Figure 6A). Moreover, mice fed a HFD supplemented with transgenic grain (RS18) had higher *Sirt1* expression in the brain, liver, skeletal muscle and adipose tissues. Among these tissues, *Sirt1* expression in the liver of the RS18-fed mice was significantly increased in comparison to that observed in the control mice fed a HFD alone (Figure 6B). A previous study reported that glucose and blood cholesterol levels were reduced in *Sirt1* transgenic mice [25]. Thus, these results suggest that treatment with resveratrol-enriched transgenic grains may improve metabolic syndrome and related diseases associated with the disturbance of hepatic lipid metabolism and of glucose and lipid homeostasis by upregulating *Sirt1* expression.

### Conclusions

After the etiological agent of the French Paradox was identified as resveratrol [26], the creation of transgenic cereal plants that accumulate resveratrol in their grains has been a major research objective. Although transgenic cereal plants have been produced with the aim of accumulating resveratrol in their grains, resveratrol was only detected at low levels in the leaves and stems of the previously created transgenic plants [19]. In this study, we report the first successful creation of rice with resveratrol-enriched grains, using the approach of validating the expression of the transgene at each step. Because the resveratrol-enriched rice was created using a rice variety with endogenous anti-metabolic syndrome characteristics, it has more potent anti-metabolic syndrome activity than resveratrol itself due to synergistic effects and can be used to treat and prevent metabolic syndrome and related diseases. Both the severity and prevalence of metabolic syndrome and related diseases, such as obesity, cardiovascular diseases, and diabetes, among many others, are currently more serious in developing countries than in developed countries. Moreover, because access to medical care is more limited in developing countries, these disorders are a more serious problem. We believe that our resveratrol-enriched rice will be an excellent alternative for the management of metabolic syndrome and related diseases in both developed and developing countries.

## Materials and Methods

### Plant Materials

The leaves and developing pods of the peanut cultivar Palkwang were used for total RNA isolation and resveratrol measurements. Seven-week-old leaves of the wild-type cultivar Dongjin and transgenic Dongjin rice plants were used for molecular characterization. Eight-week-old leaves and mature grains of rice were used to determine the levels of resveratrol and piceid.

### Cloning of AhSTS1 cDNA

Total RNA was isolated from developing peanut pods 40 days after flowering using TRIzol reagent (Invitrogen, Carlsbad, CA). The total RNA was reverse-transcribed with oligo-dT primers and the First Strand cDNA Synthesis Kit (Roche). An *STS* cDNA was cloned using RT-PCR of the first strand cDNA. Gene-specific primers (5′-ATGGTGTCTGTGAGTGGAATTC-3′ and 5′-CGTTATATGGCCACACTGC-3′) were designed based on the genomic DNA sequence of the *A. hypogaea STS* gene (GenBank accession no. AF227963) to encompass the complete coding sequence.

### Determination of AhSTS1 Activity

The 4CL enzyme converts *p*-coumaric acid into *coumaroyl* **-** *CoA by* coupling it with coenzyme A. Subsequently, three malonyl-CoA units are added to *coumaroyl* **-** *CoA* by STS with a loss of carbon dioxide, which results in the production of resveratrol [9], [10]. *AhSTS1* was amplified from cDNA using the specific primers 5′-GGATCCATGGTGTCTGTGAGTG-3′ and 5′-CTCGAGTATGGCCACACTGCGGAG-3′. The *At4CL2* gene (GenBank accession no. NM113019) was also amplified using RT-PCR from *Arabidopsis* leaf RNA using the gene-specific primers 5′-GGATCCATGACGACACAAGATGTGATAG-3′ and 5′- CTCGAGGTTCATTAATCC ATTTGCTAGT-3′ (the substitutions required to create *Bam*HI and *Xho*I restriction sites are underlined). The amplified fragments of *AhSTS1* and *At4CL2* were cloned into pET28a, a plasmid carrying a kanamycin resistance marker, and the pMAL-c2x vector, which harbors an ampicillin marker. The *AhSTS1* and *At4CL2* coding sequences were inserted in frame with the His and MBP (maltose-binding protein) carboxyl terminal tags, respectively. The plasmids containing each *AhSTS1* and *At4CL2* gene were cotransformed into BL21 *E. coli* for the induction of protein expression [27]. Finally, *E*. *coli* cells carrying the resistance genes for kanamycin and ampicillin were selected. The cells were grown in LB supplemented with 100 µg/mL of kanamycin and ampicillin at 37°C. Protein expression was induced at OD_600_ = 0.5 by adding 1 mM isopropyl *β*-D-thiogalactopyranoside (IPTG). After 24 and 48 h, the cells were harvested by centrifugation and resuspended in lysis buffer (50 mM NaH_2_PO_4_, 300 mM NaCl, and 10 mM imidazole). After sonication, the samples were subjected to SDS-PAGE for western blot analysis.

To examine resveratrol production using the recombinant proteins, *E*. *coli* cells carrying both genes were grown in 2XYT medium (10 g/L yeast extract, 16 g/L tryptone, 5 g/L NaCl) supplemented with 5 mM *p*-coumaric acid (C9008, Sigma) and 0.1 mM IPTG at 28°C. After 48 h of incubation, 1 mL of the culture medium was centrifuged at 13,000 rpm for 15 min. The supernatant was transferred to a new tube, and 50 µL 1 N hydrochloric acid was added to adjust the pH to 9.0. These samples were stored overnight at −20°C. The tubes were thawed at room temperature, and resveratrol was isolated with two extractions of equal volumes of ethyl acetate, dried under nitrogen gas, and then resuspended in 100 µL of methanol. All of the samples were stored at −20°C until they were used for the resveratrol content analysis [10], [28].

### Binary Vector Construction and Rice Transformation

To overexpress *AhSTS1* in rice, the binary vector pSB22 was constructed by inserting an expression cassette encoding the maize *Ubi1* promoter [13], multiple cloning sites (*Bam*HI, *Sma*I, and *Sac*I), and the *nopaline synthase* (*Nos*) terminator into the *Hind*III and *Eco*RI sites of the pCAMBIA3300 vector carrying the herbicide resistance *bialaphos* (*bar*) gene. Subsequently, the *AhSTS1* cDNA was inserted between the *Bam*HI and *Sac*I site under the control of the *Ubi1* promoter. The resulting plasmid was designated pSB2220. This construct was introduced into rice plants using *Agrobacterium*-mediated transformation [29]. Three-week-old calli derived from the mature seeds of the Dongjin *japonica* rice variety were cocultivated with the *A. tumefaciens* strain LBA4404 carrying pSB2220. After 3–4 weeks, transgenic calli were selected on N6 medium containing 5 mg/L phosphinothricin (PPT) and 250 mg/L cefotaxime. The transgenic plants were regenerated on MS media supplemented with 0.1 mg/L NAA, 2 mg/L kinetin, 2% sorbitol, 3% sucrose, 1.6% phytagar, 5 mg/L PPT, and 250 mg/L cefotaxime. The plants were grown in a greenhouse with a 12 h photoperiod.

### Southern Blot and RT-PCR Analysis

Approximately 3 µg of genomic DNA from the transgenic plants was digested with *Bam*HI and then subjected to electrophoresis on a 0.8% agarose gel. The DNA was transferred onto a Hybond N+ nylon membrane, and hybridization was performed using a [α-^32^P] dCTP-labeled gene-specific probe according to the standard procedures for high-stringency hybridization conditions. The blot was hybridized in a solution containing 0.5 M sodium phosphate (pH 7.2), 1 mM EDTA, 1% (w/v) BSA, and 7% (w/v) SDS for 20 h at 60°C. First-strand cDNAs, prepared from harvested leaf samples, were used in the RT-PCR reactions with gene-specific primers and control primers for *OsUBQ5*. The *AhSTS1*-specific primers were 5′-ATGGTGTCTGTGAGTGGAATTC-3′ and 5′-CGTTATATGGCCACACTGC-3′, and the *OsUBQ5*-specific primers were 5′-GACTACAACATCCAGAAGGAGTC-3′ and 5′-TCATCTAATAACCAGTTCGATTTC-3′.

### Quantification of Resveratrol and Piceid

The resveratrol and piceid levels in the transgenic rice were determined by HPLC (ACQUITY UPLC, Waters, Milford, MA) using an instrument equipped with a UV-spectrophotometer at 308 nm (ACQUITY TUV, Waters). The results were calculated using Empower software (Waters). Chromatographic separation was accomplished by injecting 1 µL of the samples onto an ACQUITY UPLC BEH-C18 1.7 µm column (2.1 mm×100 mm, Waters) at a ﬂow rate of 0.4 mL/min. The mobile phase was 10 to 90% acetonitrile (ACN). A gradient elution was conducted as follows: 0 min, 10% ACN; 1.54 min, 10% ACN; 10 min, 15% ACN; 22 min, 25% ACN; 22.4 min, 90% ACN; and 25 min, 90% ACN. The column was then re-equilibrated with 10% ACN for 5 min prior to the next injection. The calibration curves were obtained using a weighted linear regression of the peak areas against known concentrations (0.5, 1, 2, 5 and 10 µg/mL of each) of resveratrol and piceid. The weights were obtained from a smoothed estimate of the within-triplicate standard deviation of each sample [30]. The correlations of each calibration ranged from 0.98922 to 0.99917.

The HPLC fraction was eluted and further verified using GC-MS analysis with the 6890/5973N GC/MS system (Agilent Technologies) equipped with an Rtx-5MS capillary column (30 mm×0.25 mm I.D., 0.25 µm film thickness, *Restek*, Germany). The fractions were dried, resolubilized in 10 µL of methoxyamine hydrochloride, resolved in pyridine (40 mg/mL), and incubated at 30°C for 90 min. Then, 90 µL of N-methyl-N-(trimethylsilyl)trifluoroacetamide was added, and the samples were incubated at 37°C for 30 min. The resveratrol standard was prepared by solubilizing 20 µg of the compound in the same way. The initial column temperature was 80°C for 5 min, followed by a 5°C/min ramp to 300°C. Sample volumes of 1 µL were injected with a split ratio of 25∶1 using an autosampler system. The interface and ion source temperatures were set to 250°C. The resveratrol in the sample was identified by comparing the MS spectrum to the standard.

### Glucosyltransferase Activity Assay

The enzymatic activity of glucosyltransferase was measured using a previously described method [18]. Briefly, to extract the total protein, 2 g of leaves or seeds were collected from transgenic Dongjin rice and wild-type Dongjin rice. The samples were ground to a fine powder in liquid nitrogen and suspended with extraction buffer [500 mM Tris-HCl, (pH 8.0), 5 mM sodium metabisulfite, 10% glycerol, 1% PVP-40 (polyvinyl polypyrrolidone), 1 mM phenylmethyl sulfonyl fluoride, 0.1% β-mercaptoethanol, and 10% insoluble PVP]. The slurries were filtered through two layers of nylon mesh (20 µm) followed by centrifugation at 13,000 rpm for 10 min at 4°C. The protein concentration of the supernatant was determined using the Bradford reagent (BioRad, Hercules, CA). One milligram of total protein was used for the glucosyltransferase activity assay.

Each reaction mixture contained resveratrol (1 µg/mL) and rice protein extract (1 mg) in 140 µL of reaction buffer (100 mM Tris, pH 9.0). The enzyme reaction was initiated by adding 10 µL of 25 mM uridine diphosphate glucose (UDPG). Each reaction was incubated at 30°C for 30 min and terminated by the addition of 150 µL of absolute methanol. The products of the enzyme reaction were extracted twice with equal volumes of t*richloroacetic acid (*TCA) and dried under nitrogen gas. The dried residues were resuspended in 100 µL methanol. All of the samples were filtered through a 0.45 µm nylon filter after mixing with the same volume of 20% ACN for HPLC analysis. The control reactions without total protein extract or UDPG did not yield any detectable piceid.

### Animal Care and Diets

All of the procedures performed with animals were in accordance with established guidelines and were reviewed and approved by the Ethics Committee of Chonbuk National University Laboratory Animal Center. C57BL/6 female mice were purchased from Joongang Experimental Animal Co. (Seoul, Korea) at six weeks of age. The mice were housed at 10 animals per cage, with food (10% kcal as fat; D12450B; Research Diets Inc., New Brunswick, NJ) and water available ad libitum unless otherwise stated. They were maintained under a 12 h light/12 h dark cycle at a temperature of 22°C and humidity of 55±5%. After one week of acclimation, the animals were provided with a high-fat diet (HFD) containing 45% kcal as fat (D12451, Research Diets Inc.) for 12 weeks to induce metabolic syndrome and related diseases. After 12 weeks on the HFD, a total of 100 mice were randomly divided into the following groups: HFD diet (CTL), HFD supplemented with resveratrol (Resv), HFD in which the corn starch and sucrose were replaced with Dongjin rice (DJ), HFD in which half of the corn starch and sucrose were replaced with resveratrol rice (RS18-half); and HFD in which the corn starch and sucrose were replaced with resveratrol rice (RS18) (Table S3).

### In vivo Efficacy Assay

The blood glucose and lipid levels were measured at 0, 4, 8, and 12 weeks during treatment. The food consumption of each mouse group was regularly monitored. Blood samples were drawn from the tail after 5 h of fasting, and blood-glucose measurements were taken using an Accu-check Glucometer (Roche Diagnostic Corporation, Indianapolis, IN). The serum was separated by centrifuging at 13,000 rpm for 10 min and immediately stored at −20°C until assayed. The triglycerides, total cholesterol, and HDL cholesterol levels in the serum were quantitatively determined using an enzymatic colorimetric method (Asan Pharm., Yongin, Korea) [31]. The LDL cholesterol levels were calculated using the Friedewald equation [(LDL) = (T-CHO) – (HDL) – (TG)/5].

The body weights were measured at 0, 4, 8, and 12 weeks after treatment. For fat analysis, the total body fat was determined by high-resolution *in vivo* micro-CT (Skyscan 1076; SkyScan, Konitch, Belgium) with a high resolution CCD/phosphor screen detector. Before CT, the mice were anesthetized with zoletil and rumpun (4∶1) and placed on a radio-transparent mouse bed in a supine position with the caudal end closest to the micro-CT. The hind legs were extended and held in place with clear tape to ensure the correct anatomical position. Micro-CT images of the abdomen were captured at the level of the L1–L5 inter-vertebral disks, and the total fat, visceral fat and subcutaneous fat areas were analyzed using CTan Ver.1.10, Skyscan software (Skyscan).

### Determination of the Sirt1 Protein Level

Transgenic rice grains were extracted with 70% EtOH under ultrasonic conditions for 1.5 h. After repeating this process three times, the extracts were evaporated and then freeze-dried with a yield of 8.9%. SH-SY5Y cells were seeded at approximately 1×10^6^ cells in 60 mm culture dishes. After 24 h, the cells were treated with 70% ethanol extracts of transgenic grains (50 and 100 µg/mL) or resveratrol (100 µM) for 24 h. Six-week-old female C57BL/6 mice were randomly assigned to the control and transgenic rice groups. The control group was fed a HFD alone for 18 months. The transgenic rice group was fed a HFD with RS18 transgenic grain for 18 months. The organs assayed included the brain, liver, skeletal muscle and adipose tissues harvested from the mice. The cells and tissues were lysed in cold lysis buffer (0.1% SDS, 150 mM NaCl, 1% NP-40, 0.02% sodium azide, 0.5% sodium deoxycholate, 100 µg/mL PMSF, 1 µg/mL aprotinin, and phosphatase inhibitor in 50 mM Tris-HCl, pH 8.0). The levels of Sirt1 were determined by western blot analysis using an anti-Sirt1 antibody (Santa Cruz Biotechnology, Santa Cruz, CA). Briefly, 30 µg of protein was separated by SDS-PAGE (8% acrylamide gel) and transferred to a nitrocellulose membrane. The membrane was blocked with 5% non-fat skim milk in Tris-buffered saline with Tween-20 and incubated overnight with the primary antibody at 4°C. The membranes were then incubated with the secondary antibody for 1 h at room temperature. The membranes were developed using ECL reagents.

## Supporting Information

Figure S1 **Comparison of the deduced amino acid sequence of AhSTS1 and previously identified STS protein sequences.** These proteins contain conserved domain regions, such as the malonyl-CoA binding sites, a dimer interface, and active sites, which are indicated by _*_, •, and ▴, respectively. The black boxes indicate identical or conserved residues.
(TIF)

Figure S2 **Northern blot analysis of total RNA isolated from peanut leaves and pods.** The pods were collected during the early (1), middle (2), and late (3) stages of development. The *AhSTS1* cDNA was used as a probe. Strong signals were only observed in the early and middle stages of the developing peanut pods. Ethidium bromide staining of the rRNAs demonstrated equal RNA loading.
(TIF)

Figure S3 **Western blot analysis of the recombinant AhSTS1 and At4CL2 proteins.** The *AhSTS1* and *At4CL2* genes were expressed to produce fusion proteins containing a His6-tag or an MBP-tag, respectively. Total proteins were prepared from *E*. *coli* cells carrying *AhSTS1* or *At4CL2* at 24 and 48 h after adding 1 mM isopropyl *β*-D-thiogalactopyranoside (IPTG) and hybridized with rabbit anti-His6 and anti-MBP serum. AhSTS1-His6, 60 kDa; 4CL2-MBP, 103 kDa.
(TIF)

Figure S4 **GC-MS analysis of the eluted resveratrol fraction.** The MS spectrum of the resveratrol standard (A) is identical to that of the HPLC peak fraction (B). The arrows indicate the position of resveratrol.
(TIF)

Table S1 **The major agronomic characteristics of wild-type Dongjin rice and the** ***AhSTS1*** **transgenic rice line RS18.**
(DOCX)

Table S2 **The resveratrol content in unpolished and polished grains of the transgenic rice line RS18.**
(DOCX)

Table S3 **The formulation of the diets (g).**
(DOCX)
