# Supplementary figures and images for: Creation of Resveratrol-Enriched Rice for the Treatment of Metabolic Syndrome and Related Diseases
Source: PLoS One. 2013 Mar 4;8(3):e57930. doi: 10.1371/journal.pone.0057930 (PMC3587571; doi:10.1371/journal.pone.0057930)

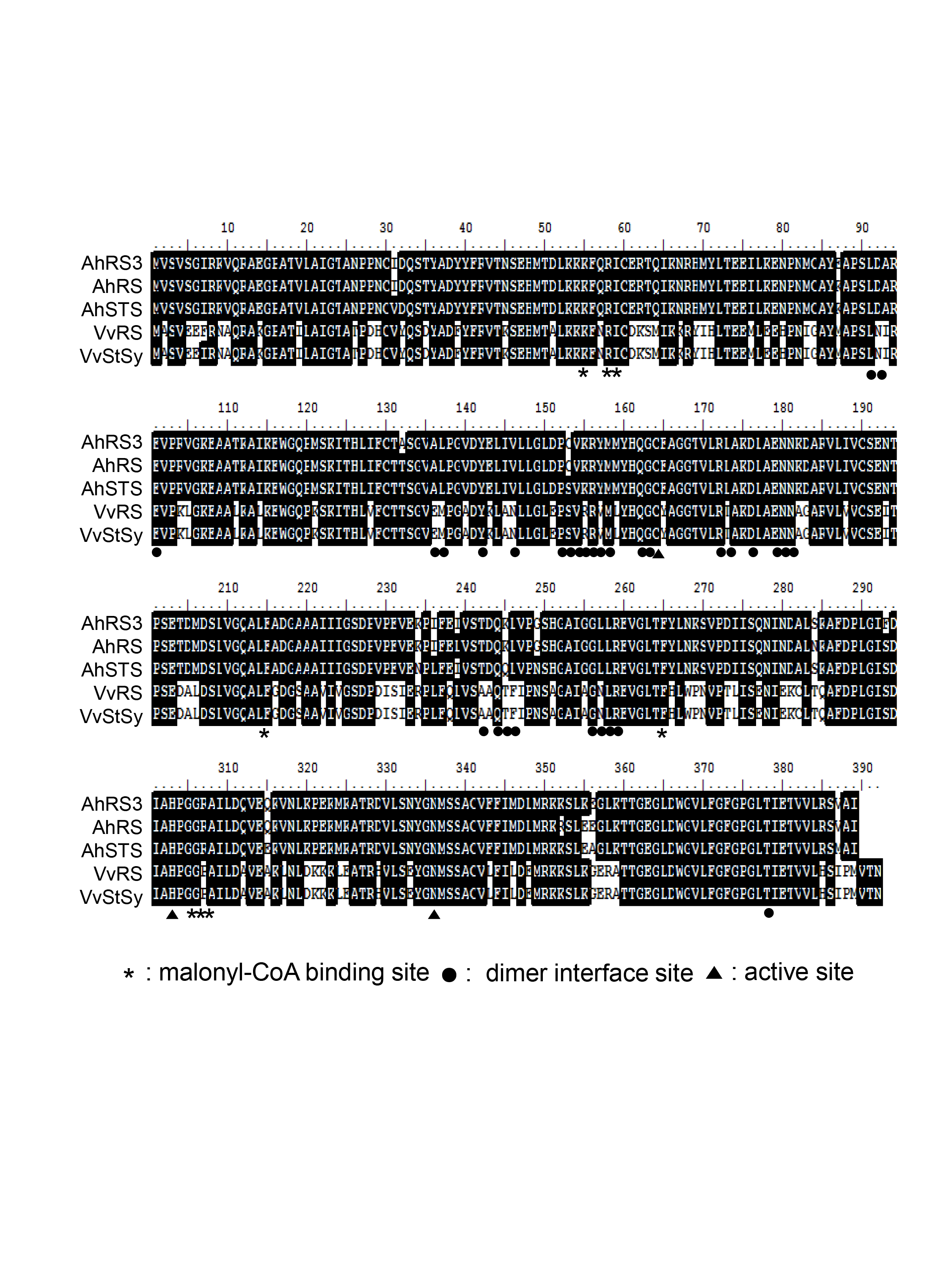

Supplement: Figure S1 — Comparison of the deduced amino acid sequence of AhSTS1 and previously identified STS protein sequences. These proteins contain conserved domain regions, such as the malonyl-CoA binding sites, a dimer interface, and active sites, which are indicated by *, •, and ▴, respectively. The black boxes indicate identical or conserved residues. (TIF) [file pone.0057930.s001.tif]

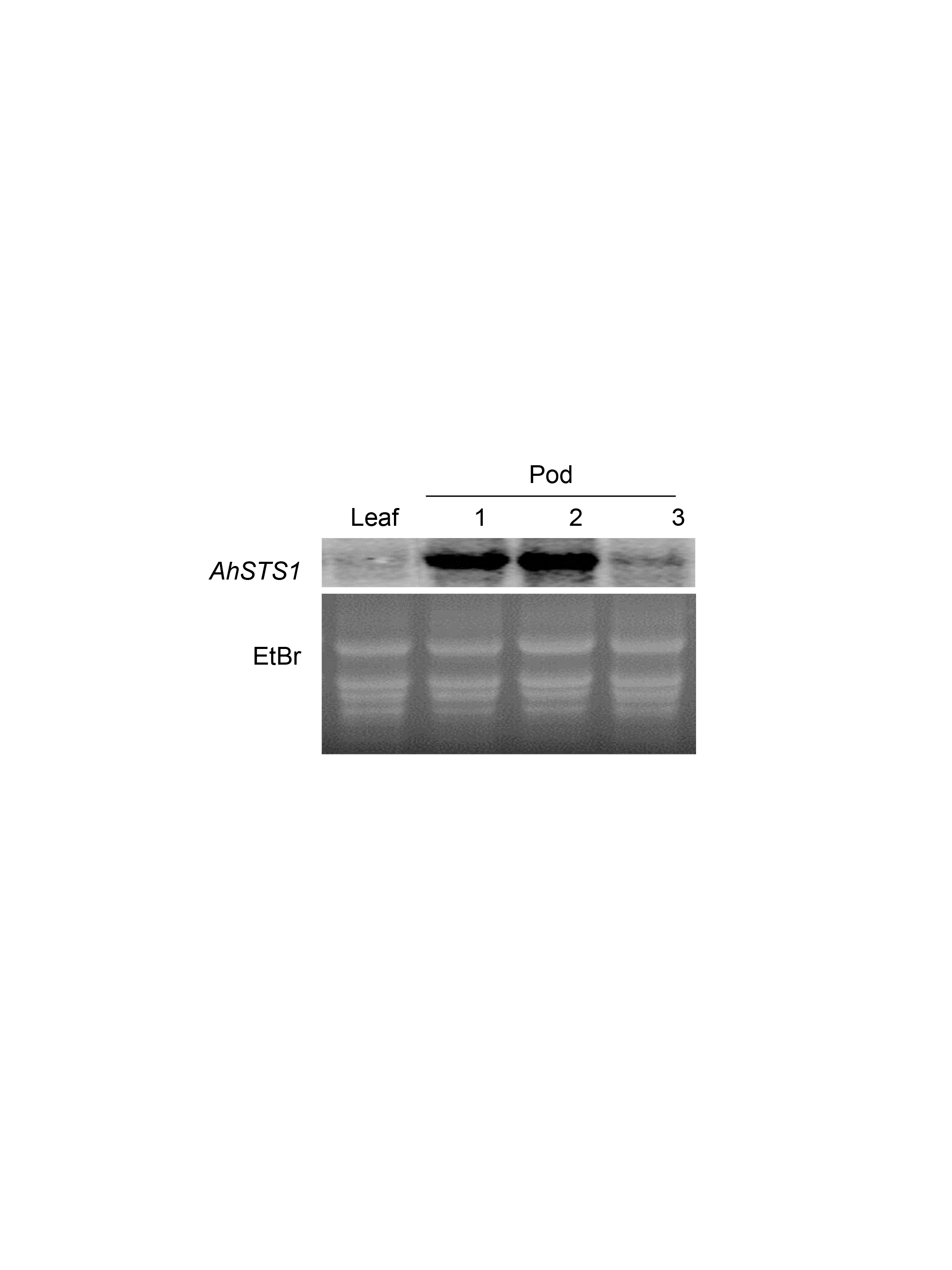

Supplement: Figure S2 — Northern blot analysis of total RNA isolated from peanut leaves and pods. The pods were collected during the early (1), middle (2), and late (3) stages of development. The AhSTS1 cDNA was used as a probe. Strong signals were only observed in the early and middle stages of the developing peanut pods. Ethidium bromide staining of the rRNAs demonstrated equal RNA loading. (TIF) [file pone.0057930.s002.tif]

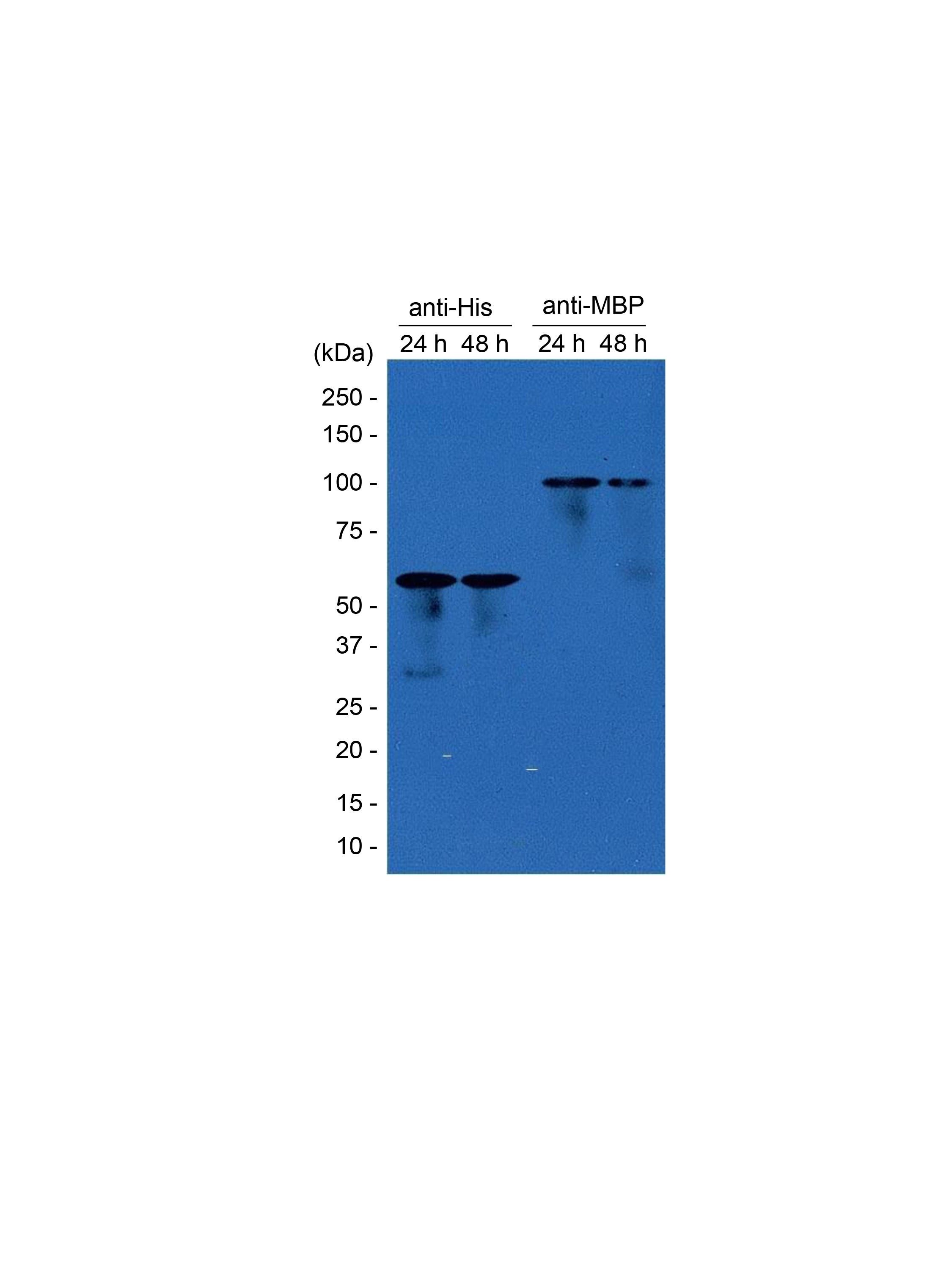

Supplement: Figure S3 — Western blot analysis of the recombinant AhSTS1 and At4CL2 proteins. The AhSTS1 and At4CL2 genes were expressed to produce fusion proteins containing a His6-tag or an MBP-tag, respectively. Total proteins were prepared from E. coli cells carrying AhSTS1 or At4CL2 at 24 and 48 h after adding 1 mM isopropyl β-D-thiogalactopyranoside (IPTG) and hybridized with rabbit anti-His6 and anti-MBP serum. AhSTS1-His6, 60 kDa; 4CL2-MBP, 103 kDa. (TIF) [file pone.0057930.s003.tif]

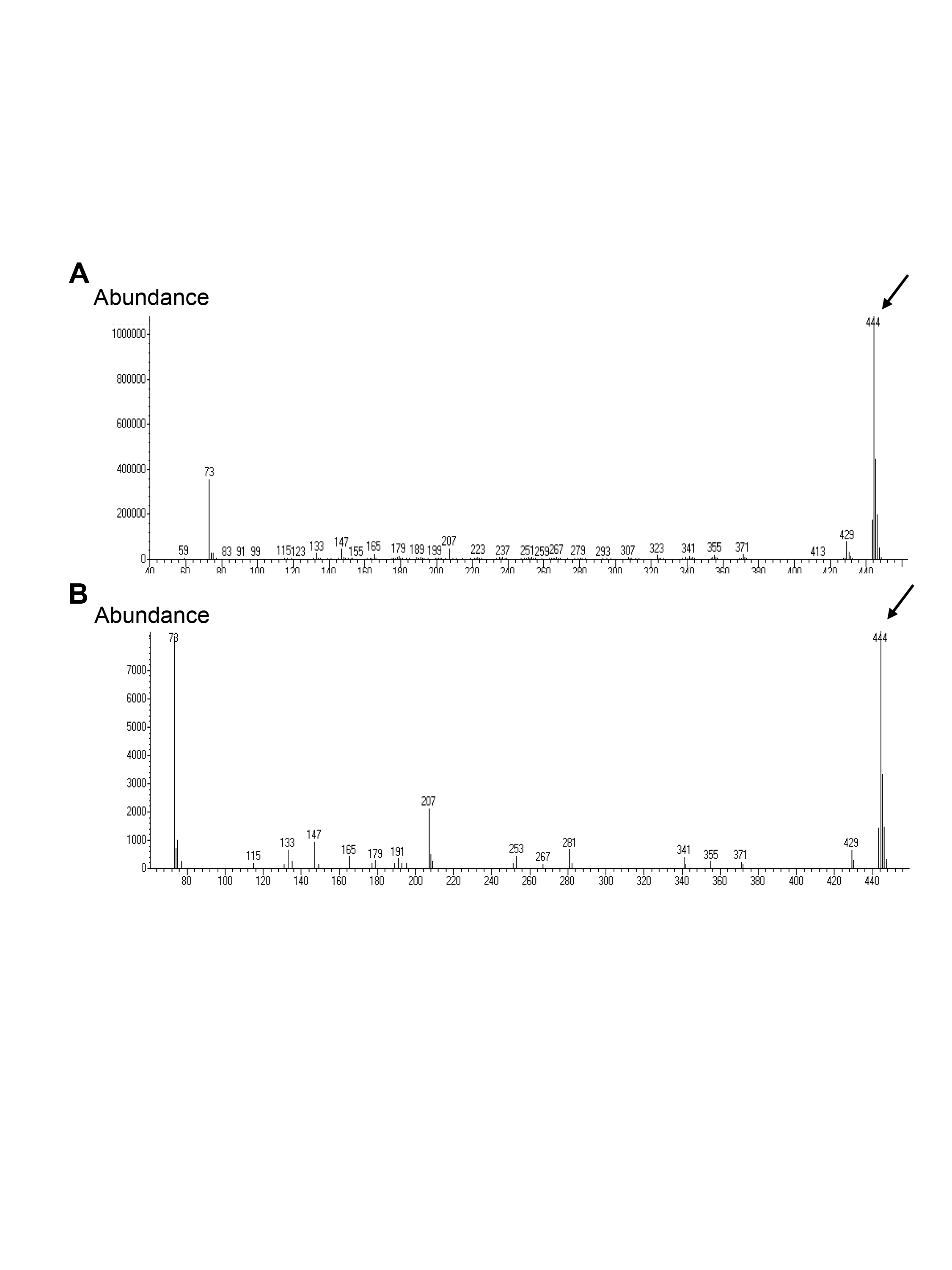

Supplement: Figure S4 — GC-MS analysis of the eluted resveratrol fraction. The MS spectrum of the resveratrol standard (A) is identical to that of the HPLC peak fraction (B). The arrows indicate the position of resveratrol. (TIF) [file pone.0057930.s004.tif]
