# Supplementary material for: Creation of Resveratrol-Enriched Rice for the Treatment of Metabolic Syndrome and Related Diseases
Source: PLoS One. 2013 Mar 4;8(3):e57930. doi: 10.1371/journal.pone.0057930 (PMC3587571; doi:10.1371/journal.pone.0057930)
Supplement: Table S1 — The major agronomic characteristics of wild-type Dongjin rice and the AhSTS1 transgenic rice line RS18. (DOCX) [file pone.0057930.s005.docx]

**Table S1. The major agronomic characteristics of wild-type Dongjin rice and the *AhSTS1* transgenic rice line RS18.**

| Lines | Heading date | Culm length (cm) | Spikelets /panicle | Ripened grain  (%) | 1,000-grain weight (g) | Brown/ rough rice  (%) | Polished white rice  (kg/10 a) |
| --- | --- | --- | --- | --- | --- | --- | --- |
| Dongjin | Aug 15 | 86 | 89 | 95.6 | 23.9 | 82.3 | 485 |
| RS18 | Aug 17 | 80 | 83 | 91.9 | 21.5 | 82.5 | 423 |
