# Supplementary material for: Creation of Resveratrol-Enriched Rice for the Treatment of Metabolic Syndrome and Related Diseases
Source: PLoS One. 2013 Mar 4;8(3):e57930. doi: 10.1371/journal.pone.0057930 (PMC3587571; doi:10.1371/journal.pone.0057930)
Supplement: Table S2 — The resveratrol content in unpolished and polished grains of the transgenic rice line RS18. (DOCX) [file pone.0057930.s006.docx]

**Table S2. The resveratrol content in unpolished and polished grains of the transgenic rice line RS18.**

| Grain type | Resveratrol (µg/g) |
| --- | --- |
| Unpolished brown grain | 1.9 ± 0.5 |
| Polished white grain | 1.7 ± 0.4 |
