# Supplementary material for: Creation of Resveratrol-Enriched Rice for the Treatment of Metabolic Syndrome and Related Diseases
Source: PLoS One. 2013 Mar 4;8(3):e57930. doi: 10.1371/journal.pone.0057930 (PMC3587571; doi:10.1371/journal.pone.0057930)
Supplement: Table S3 — The formulation of the diets (g). (DOCX) [file pone.0057930.s007.docx]

**Table S3. The formulation of the diets (g).**

| Ingredient | CTL (D12451) | Resv | DJ | RS18-half | RS18 |
| --- | --- | --- | --- | --- | --- |
| Casein, 80 Mesh | 200 | 200 | 200 | 200 | 200 |
| L-Cystine | 3 | 3 | 3 | 3 | 3 |
| **Corn Starch** | 72.8 | 72.8 | **0** | **36.4** | **0** |
| Maltodextrin 10 | 100 | 100 | 100 | 100 | 100 |
| **Sucrose** | 172.8 | 172.8 | **0** | **86.4** | **0** |
| Cellulose, BW200 | 50 | 50 | 50 | 50 | 50 |
| Soybean Oil | 25 | 25 | 25 | 25 | 25 |
| Lard | 177.5 | 177.5 | 177.5 | 177.5 | 177.5 |
| Mineral Mix | 10 | 10 | 10 | 10 | 10 |
| Dicalcium Phosphate | 13 | 13 | 13 | 13 | 13 |
| Calcium Carbonate | 5.5 | 5.5 | 5.5 | 5.5 | 5.5 |
| Potassium Citrate | 16.5 | 16.5 | 16.5 | 16.5 | 16.5 |
| Vitamin Mix | 10 | 10 | 10 | 10 | 10 |
| Choline Bitartrate | 2 | 2 | 2 | 2 | 2 |
| FD&C Red Dye #40 | 0.05 | 0.05 | 0.05 | 0.05 | 0.05 |
| **Resveratrol** | 0 | **0.00047** | 0 | 0 | 0 |
| **Dongjin Rice** | 0 | 0 | **245.6** | 0 | 0 |
| **RS18 Rice** | 0 | 0 | 0 | **122.8** | **245.6** |
| Total | 858.15 | 858.15 | 858.15 | 858.15 | 858.15 |

* Deviations from the control HFD (D12451) are indicated with bold.

After manufacturing the diets, we reconfirmed the quantity of resveratrol in each diet using HPLC before feeding the animals.
